# Clinicopathological and molecular features of responders to nivolumab for patients with advanced gastric cancer

**DOI:** 10.1186/s40425-019-0514-3

**Published:** 2019-01-31

**Authors:** Saori Mishima, Akihito Kawazoe, Yoshiaki Nakamura, Akinori Sasaki, Daisuke Kotani, Yasutoshi Kuboki, Hideaki Bando, Takashi Kojima, Toshihiko Doi, Atsushi Ohtsu, Takayuki Yoshino, Takeshi Kuwata, Akihito Tsuji, Kohei Shitara

**Affiliations:** 1 Department of Gastroenterology and Gastrointestinal Oncology, National Cancer Center Hospital East, 6-5-1 Kashiwanoha, Kashiwa, 277-8577 Japan; 2 Graduated School of Medicine, Kagawa University, Takamatsu, Japan; 3 Department of Pathology and Clinical Laboratories, National Cancer Center Hospital East, Kashiwa, Japan

**Keywords:** Nivolumab, PD-1 inhibitor, Predictive factor, Gastric cancer, Responders

## Abstract

**Background:**

Clinicopathological and molecular features of responders to nivolumab for advanced gastric cancer (AGC) are not well understood.

**Methods:**

Patients (pts) with AGC who were treated with nivolumab after two or more chemotherapy regimens in a single institution from September 2017 to May 2018 were enrolled in this study. PD-L1 expression in tumor cells (TC) and mismatch repair (MMR) were analyzed by immunohistochemistry. Epstein-Barr virus (EBV) was detected by in situ hybridization. Cancer genome alterations were evaluated by a next-generation sequencing-based panel. High tumor mutation burden (TMB) was defined as more than 10 mutations/megabase.

**Results:**

A total of 80 pts were analyzed in this study. Tumor response was evaluated in 72 pts with measurable lesions and 14 pts (19%) had an objective response. Overall response rate (ORR) was significantly higher in pts with ECOGPS 0 in those with PS 1 or 2, MMR-deficient (MMR-D) in those with MMR-proficient (MMR-P), PD-L1+ in TC in those with PD-L1- in TC and *PIK3CA* mutation in those with *PIK3CA* wild-type. ORR was 31% in pts with at least one of the following factors; MMR-D, high TMB, EBV+ and PD-L1+ in TC vs. 0% in those without these factors. Progression-free survival was significantly longer in pts with PS 0 than in those with PS 1 or 2, MMR-D than in those with MMR-P, and PD-L1+ in TC than in those with PD-L1- in TC.

**Conclusions:**

Some features were associated with favorable response to nivolumab for AGC. Combining these features might be useful to predict efficacy.

**Electronic supplementary material:**

The online version of this article (10.1186/s40425-019-0514-3) contains supplementary material, which is available to authorized users.

## Introduction

Recently, blockade of immune checkpoint molecules with monoclonal antibodies has emerged as a promising strategy in several malignancies [1–6]. Programmed death 1 (PD-1), which belongs to the CD28 family of proteins, is a negative costimulatory receptor expressed on the surfaced of activated T cells [7]. The binding of PD-1 and its ligands, PD-L1 and PD-L2 in tumor or immune cells, can inhibit a cytotoxic T-cell response, which leads tumor cells to escape from immune surveillance [7]. Accordingly, blockade of this interaction restores the antitumor activity of T cells [7]. Clinical trials of anti-PD-1/PD-L1 monoclonal antibodies have shown durable anti-tumor response and improved overall survival in several malignancies [1–6].

A phase III ATTRACTION-2 trial of nivolumab, a fully human IgG4 monoclonal antibody (mAb) against PD-1, for patients (pts) with advanced gastric cancer (AGC) after two or more previous line chemotherapies showed a survival benefit, leading to the approval of nivolumab for AGC in Japan [8]. Exploratory analysis of ATTRACTION-2 suggested a survival benefit of nivolumab regardless of PD-L1 expression on tumor cells, thus nivolumab have been used without any restriction by biomarkers [8].

Pembrolizumab, another PD-1 mAb, also demonstrated encouraging anti-tumor activity with acceptable safety for PD-L1 positive AGC in phase II and III trials [9, 10], where PD-L1 expression has been evaluated as combined positive score (CPS) counting both tumor cells and immune cells. A relationship between greater PD-L1 CPS and a greater treatment effect was suggested in phase II and III trials of pembrolizumab [9, 10]. ORR in pts with CPS ≥ 10, CPS ≥ 1, and CPS < 1 (PD-L1-) were 25, 16, and 2%, respectively [10]. Recently, the US Food and Drug Administration approved pembrolizumab for pts with microsatellite instability-high or mismatch repair (MMR) deficient solid tumors including AGC based on the durable response in several trials [11–13]. In addition to PD-L1 expression and MMR deficiency, a small study suggested that high tumor mutation burden (TMB) and EBV positive status were associated with response to pembrolizumab [14]. However, predictive factors of nivolumab for AGC have not yet been evaluated. Also, overlapping of several clinicopathological and molecular features have not yet been discussed in detail.

In order to establish the better selection of pts who may derive greater benefit from PD-1 blockade, we investigated clinicopathological and molecular features of responders to nivolumab for AGC.

## Patients and method

### Patients

A prospective study was performed to evaluate the efficacy of nivolumab in pts with AGC from September 2017 to May 2018 at the National Cancer Center Hospital East. The eligibility criteria were the presence of histologically proven adenocarcinoma; Eastern Cooperative Oncology Group performance status (ECOG PS) of 0–2; adequate bone marrow, hepatic, and renal function; history of previous treatment with two or more regimens and at least one treatment with nivolumab. All patients provided written, informed consent prior to participating in this observational study. The study protocol was approved by the Institutional Review Board at the National Cancer Center.

### Molecular characteristics

Molecular characteristics, such as status of human epidermal growth factor receptor 2 (HER2), PD-L1, MMR, and EBV, and genomic alterations, were analyzed with formalin-fixed paraffin-embedded tissue specimens from archival tissue samples if available. Immunohistochemistry (IHC) using a monoclonal anti-HER2 antibody (PATHWAY HER2 [4B5], Ventana, Tucson, AZ) and fluorescence in situ hybridization (FISH) using the PathVysion HER-2 probe kit (Abbott Laboratories, Abbott Park, IL) were performed to assess HER2 status, and HER2 positive was defined as IHC 3 + or IHC 2+ and FISH positive. PD-L1 IHC was performed using an anti-PD-L1 rabbit monoclonal antibody (Clone SP142 or SP263, Ventana), and PD-L1 positive in tumor cells (TC) or immune cells (IC) was defined as the presence of ≥1% of TC or IC with membrane staining. CPS, which was the number of PD-L1 staining cells (TC, lymphocytes, and macrophages) divided by the total number of viable TC multiplied by 100, was also assessed. MMR status was assessed by IHC using monoclonal antibodies for anti-mutL homolog 1 (MLH1, ES05), anti-mutS homolog 2 (MSH2, FE11), anti-postmeiotic segregation increased 2 (PMS2, EP51), and anti-mutS homolog 6 (MSH6, EP49) (Agilent Technologies, Santa Clara, CA), and tumors lacking either MLH1, MSH2, PMS2, or MSH6 expression were considered MMR-deficient (MMR-D), whereas tumors that maintained expression of MLH1, MSH2, PMS2, and MSH6 were considered MMR proficient (MMR-P). Chromogenic in situ hybridization for EBV-encoded RNA (EBER) using fluorescein-labeled oligonucleotide probes (INFORM EBER Probe, Ventana) was performed to assess EBV status [15]. All the specimens were reviewed by TK for this study. Genomic alterations were assessed using Oncomine™ Comprehensive Assay version 3 or Oncomine™ Cancer Research Panel (Thermo Fisher Scientific, Waltham, MA), which allows to detect gene mutations, copy number variants and fusions across multiple genes (Additional file 1: Table S1). TMB was defined as the number of non-synonymous mutations, including indel, per megabase (mt/Mb) of genome examined in tumor tissue. Known germline variants in dbSNP and East Asian population of 1000 Genomes or ExAC database were not counted. High TMB was defined as more than 10 mutations per megabase.

### Outcomes and statistical analysis

We assessed ORR, disease control rate (DCR), and progression-free survival (PFS). Tumor response was assessed in pts with measurable lesions according to the guidelines of the Response Evaluation Criteria in Solid Tumors version 1.1. ORR was defined as the proportion of pts with the best overall response of complete response (CR) or partial response (PR). DCR was defined as the proportion of pts with the best overall response of CR, PR, or stable disease (SD). Responder was defined as pts who achieved CR or PR, while non-responder was defined as those who showed SD or progression disease (PD). PFS was defined from the date of initiation of nivolumab to the date of disease progression or death from any cause.

Statistical comparisons of ORR according to baseline characteristics was performed using Chi-square test or Fisher’s exact test. PFS was estimated by the Kaplan–Meier method and compared according to baseline characteristics using Cox proportional hazards models and presented as hazard ratios (HRs) with 95% confidence intervals (CIs). Multivariate analysis for PFS was conducted using clinical factors which were associated with significant impact on PFS as well as potential predictive biomarkers reported in previous studies. Statistical analyses were performed using SPSS® Statistics software V21 (IBM, Armonk, NY, US). All tests were two-sided; *p* < 0.05 was considered to indicate statistical significance.

## Results

### Patient characteristics

A total of 80 pts were analyzed in this study. Baseline patient characteristics were shown in Additional file 2: Table S2. The median age was 67 (range, 25–86) years, and 61 pts (76%) were male. Forty-seven pts (59%) had an ECOG PS of 0, whereas the remaining 33 pts (41%) had a PS of 1 or 2 at the initiation of nivolumab treatment. Forty-nine pts (61%) had been treated with three or more lines of previous chemotherapies before nivolumab treatment. The most common metastatic site was the lymph node (75%), followed by the peritoneum and liver. Sixteen pts (20%) showed HER2-positive tumors. Eight pts (10%) were found to have MMR-D status, and 4 (5%) pts showed EBV-positive tumors.

### Clinicopathological and molecular features of responders to nivolumab

Of 80 pts with AGC, tumor response was evaluated in 72 pts with measurable lesions. Best responses were CR, PR, SD, and PD in 0 (0%), 14 (19%), 20 (28%), and 38 (53%), respectively, resulting in ORR of 19% and DCR of 47%.

ORR was significantly higher in pts with PS of 0 than in those with PS of 1 or 2 (30% vs. 3%, *p* < 0.01) (Table 1). There were no other clinical factors significantly associated with responders. ORR tended to be higher in pts with lymph node metastasis than in those without (24% vs. 0%, *p* = 0.05), although the differences were not statistically significant.

**Table 1:** Clinical features of responders to nivolumab

*n* = 72		All	Responder	Non-responder	ORR	*P*-value
Age	< 65	26 (36%)	8	18	31%	0.07
	≥65	46 (64%)	6	40	13%	
Gender	Male	56 (78%)	11	45	20%	0.94
	Female	16 (22%)	3	13	19%	
ECOG PS	0	43 (60%)	13	30	30%	< 0.01
	≥1	29 (40%)	1	28	3%	
Histology	Intestinal	31 (43%)	5	26	16%	0.54
	Diffuse	41 (57%)	9	32	22%	
Borrmann classification	Type4	7 (10%)	0	7	0%	0.17
	Others	65 (90%)	14	51	22%	
Number of previous chemotherapy	2	29 (40%)	8	21	28%	0.15
	≥3	43 (60%)	6	37	14%	
Site of metastasis	Lymph node	59 (82%)	14	45	24%	0.05
	Peritoneum	35 (49%)	5	30	14%	0.28
	Liver	32 (44%)	4	28	13%	0.18
	Lung	10 (14%)	1	9	10%	0.42
Number of metastatic sites	1	21 (29%)	4	17	19%	0.96
	≥2	51 (71%)	10	41	20%	

*ECOG PS* Eastern Cooperative Oncology Group performance status, *ORR* objective response rate

ORR was significantly higher in pts with MMR-D than in those with MMR-P (75% vs. 13%, *p* < 0.01), PD-L1+ in TC than in those with PD-L1- in TC (57% vs. 13%, *p* < 0.01), and *PIK3CA* mutation in those with *PIK3CA* wild-type (44% vs. 14%, *p* = 0.03) (Table 2). There were no other molecular factors significantly associated with responders. Additional file 3: Figure S1 also showed no significant relationship between TMB and response to nivolumab. ORR in pts with CPS ≥ 10, CPS ≥ 1, CPS < 1 (PD-L1-), EBV+ and high TMB were 35, 26, 0, 25, and 22%, respectively. After excluding 8 pts with MMR-D from the analysis, PS of 0 and PD-L1+ in TC were factors significantly associated with responders in 60 pts with MMR-P (Additional file 4: Table S3 and Additional file 5: Table S4).

**Table 2:** Molecular features of responders to nivolumab

	Assessed	Detected	Responder	Non-responder	ORR	*P*-value
HER2+	71	16 (23%)	1	15	6%	0.12
PD-L1+ in tumor cell	60	14 (23%)	8	6	57%	< 0.01
CPS≥10	60	17 (28%)	6	11	35%	0.17
CPS≥1	60	54 (90%)	14	40	26%	0.15
EBV+	68	4 (6%)	1	3	25%	0.82
MMR-D	68	8 (12%)	6	2	75%	< 0.01
TMB≥10	54	32 (59%)	7	25	22%	0.44
*ARID1A* mutation	52	5 (10%)	1	4	25%	0.96
*ERBB2* mutation	52	2 (4%)	0	2	0%	0.48
*KRAS* mutation	52	4 (8%)	0	4	0%	0.31
*MET* mutation	52	2 (4%)	0	2	0%	0.48
*PIK3CA* mutation	52	9 (17%)	4	5	44%	0.03
*TP53* mutation	52	28 (54%)	6	22	21%	0.66
*CCNE1* amplification	52	7 (13%)	2	5	29%	0.50
*ERBB2* amplification	52	9 (17%)	0	9	0%	0.11
*FGFR* amplification	52	3 (6%)	0	3	0%	0.38
*MDM2* amplification	52	2 (4%)	0	2	0%	0.48
*MYC* amplification	52	3 (6%)	0	3	0%	0.38

*CPS* combined positive score, *EBV* Epstein-Barr virus, *MMR-D* mismatch repair deficient, *ORR* objective response rate, *PD-L1* programmed cell death-1 ligand-1, *TMB* tumor mutation burden

Table 3 showed characteristics of pts with response to nivolumab. Among the 14 responders, 6 were MMR-D and other 8 were MMR-P. TMB was assessed in 4 MMR-D pts., and 3 of them were with high TMB (range 11.5 to 58.0). Four MMR-P responders were also associated with high TMB (range 10.1 and 15.3). One MMR-P responder was EBV+ with TMB of 7.7 and the remaining 3 MMR-P responders were PD-L1+ in TC. Among MMR-D or EBV+ pts., no EBV+ pts showed PD-L1+ in TC or CPS ≥ 10. Two patients with MMR-D without tumor response had PS of 1 or PS of 2 as well as *PIK3CA* mutations (Additional file 6: Table S5).

**Table 3:** Characteristics of patients with response to nivolumab

Age	PS	Genomic alteration			PD-L1+ in TC	CPS≥10	CPS≥1	EBV	MMR
		Mutation	Amplification	TMB/Mb					
63	0	NE	NE	NE	–	+	+	–	MMR-D
63	0	NE	NE	NE	+	+	+	–	MMR-D
66	0	*PIK3CA, TP53*	None	38.3	+	–	+	–	MMR-D
62	0	*PIK3CA*	None	11.5	–	–	+	–	MMR-D
53	1	None	None	7.7	+	+	+	–	MMR-D
79	0	*MET, PIK3CA, TP53*	None	58.0	+	–	+	–	MMR-D
64	0	*PIK3CA*	None	15.3	+	+	+	–	MMR-P
74	0	*ARID1A, TP53*	*CCNE1*	15.1	–	–	+	–	MMR-P
80	0	*TP53*	*CCNE1*	11.5	–	–	+	–	MMR-P
76	0	None	None	10.1	–	–	+	–	MMR-P
73	0	*TP53*	None	5.0	+	+	+	–	MMR-P
65	0	NE	NE	NE	+	+	+	–	MMR-P
53	0	NE	NE	NE	+	–	+	–	MMR-P
43	0	*TP53*	None	7.7	–	–	+	+	MMR-P

*CPS* combined positive score, *EBV* Epstein-Barr virus, *MMR* mismatch repair, *MMR-D* mismatch repair deficient, *MMR-P* mismatch repair proficient, *NE* not examined, *ORR* objective response rate, *PD-L1* programmed cell death-1 ligand-1, *PS* Eastern Cooperative Oncology Group performance status, *TMB* tumor mutation burden

Importantly, ORR was 31% in pts with at least one of the following factors; MMR-D, high-TMB, EBV+, and PD-L1+ in TC vs. 0% in those without these factors.

#### Progression free survival analysis

**Fig. 1:**
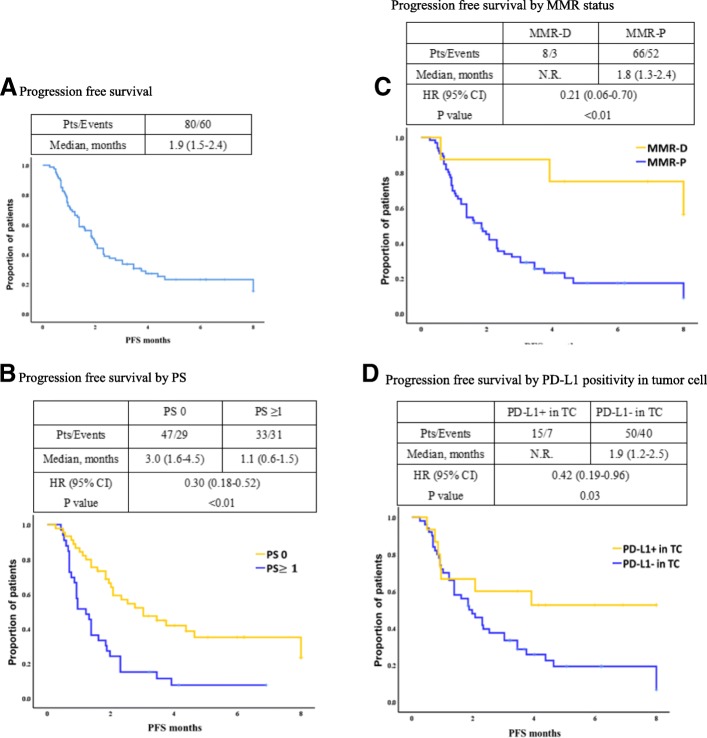
Progression free survival. **a** Progression free survival. **b** Progression free survival by PS. **c** Progression free survival by MMR status. **d** Progression free survival by PD-L1 positivity in tumor cell. MMR, mismatch repair; MMR-D, mismatch repair deficient; MMR-P, mismatch repair proficient; PD-L1, programmed cell death-1 ligand-1; PS, Eastern Cooperative Oncology Group performance status; Pts, patient

In 80 pts with AGC, the median PFS of nivolumab was 1.9 (95% CI, 1.5–2.4) months with median follow-up period of 3.8 months (range, 0.3–8.0 months) (Fig. 1a). Subgroup analysis of PFS was shown in Additional file 7: Table S6. PFS was significantly longer in pts with PS of 0 than in those with PS of 1 or 2 (median 3.0 months vs. 1.1 months, HR 0.30; 95% CI 0.18–0.52, *p* < 0.01) (Fig. 1b), MMR-D than MMR-P (median not reached vs. 1.8 months, HR 0.21; 95% CI 0.06–0.70, *p* < 0.01) (Fig. 1c), and PD-L1+ in TC than PD-L1- in TC (median not reached vs. 1.9 months, HR 0.42; 95% CI 0.19–0.96, *p* = 0.03) (Fig. 1d). In univariate analysis for PFS, PS was only clinical factor associate with PFS. Furthermore, after adjusted by PS, the HR for PFS of pts with MMR-D compared to those with MMR-P and pts with PD-L1+ in TC compared to those with PD-L1- in TC was 0.2 (95% CI 0.1–0.6; *p* < 0.01) and 0.4 (95% CI 0.2–0.9; p = 0.03), respectively (Additional file 7: Table S6). Pts with other molecular features associated with response to nivolumab in this study did not show significantly longer PFS.

## Discussion

In this study, we investigated the characteristics of responders to nivolumab for pts with AGC. To our knowledge, this is the first report to provide detailed information on clinicopathological and molecular features associated with response to nivolumab for AGC.

The results of subgroup analysis of phase II and III trials of pembrolizumab showed that better PS was associated with a higher response rate and longer overall survival [10, 11]. Consistent with these results, pts with PS of 0 had better ORR and PFS compared to those with PS of 1 or 2 in our study. Furthermore, after excluding pts with MMR-D from the analysis, PS of 0 was an only clinical factor significantly associated with responders in pts with MMR-P, suggesting that it is important to assess general condition before the initiation of PD-1 blockade for the prediction of efficacy. Although the exact explanations for the correlation between PS and clinical outcomes of PD-1 blockade were not established, pts with poor PS may not stay on treatment long enough to achieve a response.

In our analysis, PD-L1 expression in TC was significantly associated with responders to nivolumab for AGC, which was contrary to that of subgroup analysis from ATTRACTION-2 [8]. Furthermore, after excluding pts with MMR-D, impact of PD-L1 in TC was still statistically significant. Different anti-PD-L1 antibodies (SP142 or SP263) in our study from those (28–8 or 22C3) in these previous studies of nivolumab or pembrolizumab [9, 10, 14] might affect the predictive value of PD-L1 expression. Also, ORR and PFS tended to be better in pts with CPS ≥ 10 overlapping substantially with PD-L1+ in TC in our analysis; 5 of 14 responders had both CPS ≥ 10 and PD-L1+ in TC. Impact of CPS on the efficacy of PD-1 blockade will also be evaluated in the ongoing phase III KEYNOTE­062 trial (NCT02494583), which compared the efficacy of cytotoxic agents combined to pembrolizumab with that of cytotoxic agents and that of pembrolizumab monotherapy in pts with untreated AGC.

ORR was significantly higher in AGC pts with *PIK3CA* mutation in our study, which was consistent with a recent study analyzing genomic correlates of response to immune checkpoint blockade in microsatellite-stable solid tumors [16]. It is also suggested that *PIK3CA* mutation have been linked with APOBEC signatures which is highly proficient at generating DNA breaks whose repair can trigger the formation of single-strand hypermutation substrates [17]. Moreover, in gastric cancer, it has been well known that APOBEC-mutation signature and *PIK3CA* mutation were frequently observed in EBV+ pts [18]. Meanwhile, it is reported that *PIK3CA* mutation is strongly associated with the MSI molecular subgroup [19]. Among 4 responders with *PIK3CA* mutation in our study, 3 were MMR-D, and only additional one patient with MMR-P, no EBV+, and PD-L1 in TC with CPS ≥ 10 had mutation in *PIK3CA* lie in E542K, which has been reported to be associated with APOBEC signature. Thus, the predictive value of *PIK3CA* mutation alone in AGC needs further investigations. Most recently, extremely high ORR (100%) of pembrolizumab was reported in 6 pts with EBV+ AGC [14], which was inconsistent with our result showing that 1 of 4 pts with EBV+ (25%) achieved an objective response. Notably, no EBV pts in our study showed CPS ≥ 10, which was different from previous study [14]. Our pervious study showed not all EBV+ tumors showed high PD-L1 expression [15], thus both EBV status and PD-L1 expression should be evaluated in a larger cohort.

High TMB was not associated with response to nivolumab in our study, though it was notable that 4 of 8 responder pts with MMR-P had high TMB. It has been reported that the estimated TMB based on panel sequencing showed relatively high discordance compared with TMB calculated from whole exome sequencing in tumors with relatively low number of mutations [20], which might lead to the results in this study which did not show good correlation between anti-tumor response and TMB. Recently, Kim ST et al. reported that high TMB was a potential biomarker of pembrolizumab for AGC [14]. However, most pts with high TMB had MMR-D status, and not all pts with high TMB achieved an objective response [14]. Thus, precise mechanism regarding the influence of TMB to the efficacy of PD-1/PD-L1 blockade should be investigated in the near future.

Interestingly, ORR was 31% in pts with at least one of the following factors; MMR-D, high-TMB, EBV+, and PD-L1+ in TC vs. 0% in those without these factors, suggesting that pre-screening of these biomarkers might be useful to predict clinical benefit of anti-PD-1/PD-L1 blockade in AGC.

It is important to note the limitations of the present study. First, this was a single-institution study with a limited sample size. Second, we did not analyze PD-L1 expression, MMR, EBV status, and cancer genome alterations in all the pts enrolled in this study, which warrants further evaluations in a larger cohort.

In conclusion, we identified some clinicopathological and molecular characteristics associated with responders to nivolumab for pts with AGC. Combining these features might be useful for the better selection of pts who may derive greater benefit from PD-1 blockade. However, further investigations in larger cohorts are needed to confirm precise biomarkers of PD-1/PD-L1 blockade for AGC.

## Additional files

Additional file 1: **Table S1**. Gene list of the Oncomine™ Comprehensive Assay version 3. (DOCX 15 kb)

Additional file 2: **Table S2**. Patient characteristics. (DOCX 17 kb)

Additional file 3: Figure S1. Response to nivolumab by tumor mutation burden. (DOCX 22 kb)

Additional file 4: **Table S3**. Clinicopathological features of responders to nivolumab in patients with MMR-P. (DOCX 16 kb)

Additional file 5: **Table S4**. Molecular features of responders to nivolumab in patients with MMR-P. (DOCX 16 kb)

Additional file 6: **Table S5**. Characteristics of patients with MMR-D and EBV positive gastric cancer. (DOCX 18 kb)

Additional file 7: **Table S6**. Subgroup analysis of progression-free survival. (DOCX 16 kb)

## Abbreviations

AGC: Advanced gastric cancer
CIs: Confidence intervals
CPS: Combined positive score
CR: Complete response
DCR: Disease control rate
EBER: EBV-encoded RNA
EBV: Epstein-Barr virus
ECOG PS: Eastern Cooperative Oncology Group performance status
FISH: Fluorescence in situ hybridization
HER2: Human epidermal growth factor receptor 2
HR: Hazard ratio
IC: Immune cell
IHC: Immunohistochemistry
mAbs: Monoclonal antibodies
MLH1: Anti-mutL homolog 1
MMR: Mismatch repair
MMR-D: Mismatch repair deficient
MMR-P: Mismatch repair proficient
MSH2: Anti-mutS homolog 2
MSH6: Anti-mutS homolog 6
mt/MB: Mutations/megabase
ORR: Objective response rate
PD: Progressive disease
PD-1: Anti-programmed death-1
PD-L1: Programmed death-ligand 1
PD-L2: Programmed death-ligand 2
PFS: Progression-free survival
PMS2: Anti-postmeiotic segregation increased 2
PR: Partial response
pts.: Patients
SD: Stable disease
TC: Tumor cell
TMB: Tumor mutation burden
