# Supplementary material for: Clinicopathological and molecular features of responders to nivolumab for patients with advanced gastric cancer
Source: J Immunother Cancer. 2019 Jan 31;7:24. doi: 10.1186/s40425-019-0514-3 (PMC6357506; doi:10.1186/s40425-019-0514-3)
Supplement: Supplementary file 1 — Table S1. Gene list of the Oncomine™ Comprehensive Assay version 3. (DOCX 15 kb) [file 40425_2019_514_MOESM1_ESM.docx]

Table S1. Gene list of the Oncomine™ Comprehensive Assay version 3

| **Hotspot genes** | **Full-length genes** | **Copy number genes** | **Gene fusions (inter- and intragenic)** |
| --- | --- | --- | --- |
| *AKT1* | *ATM* | *AKT1* | *ALK* |
| *ALK* | *BAP1* | *AR* | *AXL* |
| *AR* | *BRCA1* | *CCND1* | *BRAF* |
| *ARAF* | *BRCA2* | *CCNE1* | *EGFR* |
| *BRAF* | *CDKN2A* | *CDK4* | *ERBB2* |
| *BTK* | *FBXW7* | *CDK6* | *ERG* |
| *CBL* | *MSH2* | *EGFR* | *ETV1* |
| *CDK4* | *NF1* | *ERBB2* | *ETV4* |
| *CHEK2* | *NF2* | *FGFR1* | *ETV5* |
| *CSF1R* | *NOTCH1* | *FGFR2* | *FGFR1* |
| *CTNNB1* | *PIK3R1* | *FGFR3* | *FGFR2* |
| *DDR2* | *PTCH1* | *FGFR4* | *FGFR3* |
| *EGFR* | *PTEN* | *FLT3* | *NTRK1* |
| *ERBB2* | *RB1* | *IGF1R* | *NTRK3* |
| *ERB83* | *SMARCB1* | *KIT* | *PDGFRA* |
| *ERBB4* | *STK11* | *KRAS* | *PPARG* |
| *ESR1* | *TP53* | *MDM2* | *RAF1* |
| *EZH2* | *TSC1* | *MDM4* | *RET* |
| *FGFR1* | *TSC2* | *MET* | *ROS1* |
| *FGFR2* | *ARID1A* | *MYC* | *AKT2* |
| *FGFR3* | *ATR* | *MYCL* | *AR* |
| *FLT3* | *ATRX* | *MYCN* | *BRCA1* |
| *FOXL2* | *CDK12* | *PDGFRA* | *BRCA2* |
| *GATA2* | *CDKN1B* | *PIK3CA* | *CDKN2A* |
| *GNA11* | *CDKN2B* | *PPARG* | *ERB84* |
| *GNAQ* | *CHEK1* | *TERT* | *ESR1* |
| *GNAS* | *CREBBP* | *AKT2* | *FGR* |
| *HNF1A* | *FANCA* | *AKT3* | *FLT3* |
| *HRAS* | *FANCD2* | *ALK* | *JAK2* |
| *IDH1* | *FANCI* | *AXL* | *KRAS* |
| *IDH2* | *MLH1* | *BRAF* | *MDM4* |
| *JAK1* | *MRE11A* | *CCND2* | *MET* |
| *JAK2* | *MSH6* | *CCND3* | *MYB* |
| *JAK3* | *NBN* | *CDK2* | *MYBL1* |
| *KDR* | *NOTCH2* | *CDKN2A* | *NF1* |
| *KIT* | *NOTCH3* | *CDKN2B* | *NOTCH1* |
| *KNSTRN* | *PALB2* | *ESR1* | *NOTCH4* |
| *KRAS* | *PMS2* | *FGF19* | *NRG1* |
| *MAGOH* | *POLE* | *FGF3* | *NTRK2* |
| *MAP2K1* | *RAD50* | *NTRK1* | *NUTM1* |
| *MAP2K2* | *RAD51* | *NTRK2* | *PDGFRB* |
| *MAPK1* | *RAD51B* | *NTRK3* | *PIK3CA* |
| *MAX* | *RAD51C* | *PDGFRB* | *PRKACA* |
| *MED12* | *RAD51D* | *PIK3CB* | *PRKACB* |
| *MET* | *RNF43* | *RICTOR* | *PTEN* |
| *MTOR* | *SETD2* | *TSC1* | *RAD51B* |
| *MYD88* | *SLX4* | *TSC2* | *RB1* |
| *NFE2L2* | *SMARCA4* |  | *RELA* |
| *NRAS* |  |  | *RSPO2* |
| *PDGFRA* |  |  | *RSPO3* |
| *PIK3CA* |  |  | *TERT* |
| *PPP2R1A* |  |  |  |
| *PTPN11* |  |  |  |
| *RAC1* |  |  |  |
| *RAF1* |  |  |  |
| *RET* |  |  |  |
| *RHEB* |  |  |  |
| *RHOA* |  |  |  |
| *SF3B1* |  |  |  |
| *SMO* |  |  |  |
| *SPOP* |  |  |  |
| *SRC* |  |  |  |
| *STAT3* |  |  |  |
| *U2AF1* |  |  |  |
| *XPO1* |  |  |  |
| *AKT2* |  |  |  |
| *AKT3* |  |  |  |
| *AXL* |  |  |  |
| *CCND1* |  |  |  |
| *CDK6* |  |  |  |
| *ERCC2* |  |  |  |
| *FGFR4* |  |  |  |
| *H3F3A* |  |  |  |
| *HIST1H3B* |  |  |  |
| *MAP2K4* |  |  |  |
| *MDM4* |  |  |  |
| *MYC* |  |  |  |
| *MYCN* |  |  |  |
| *NTRK1* |  |  |  |
| *NTRK2* |  |  |  |
| *PDGFRB* |  |  |  |
| *PIK3CB* |  |  |  |
| *ROS1* |  |  |  |
| *SMAD4* |  |  |  |
| *TERT* |  |  |  |
| *TOP1* |  |  |  |
