# Supplementary material for: Clinicopathological and molecular features of responders to nivolumab for patients with advanced gastric cancer
Source: J Immunother Cancer. 2019 Jan 31;7:24. doi: 10.1186/s40425-019-0514-3 (PMC6357506; doi:10.1186/s40425-019-0514-3)
Supplement: Supplementary file 2 — Table S2. Patient characteristics. (DOCX 17 kb) [file 40425_2019_514_MOESM2_ESM.docx]

Table S2. Patient characteristics

|  |  | All (*n* =80) |
| --- | --- | --- |
| Age | Median (range) | 67 (25-86) |
| Gender | Male | 61 (76%) |
|  | Female | 19 (24%) |
| ECOG PS | 0 | 47 (59%) |
|  | 1 | 30 (38%) |
|  | 2 | 3 (3%) |
| Histology | Intestinal | 34 (42%) |
|  | Diffuse | 46 (58%) |
| HER2 | positive | 16 (20%) |
| EBV | positive | 4 (5%) |
| MMR | MMR-D | 8 (10%) |
| Previous gastrectomy | Yes | 29 (36%) |
| Number of previous chemotherapy | 2 | 31 (39%) |
|  | ≥3 | 49 (61%) |
| Site of metastasis | Lymph node | 60 (75%) |
|  | Peritoneum | 39 (49%) |
|  | Liver | 32 (40%) |
|  | Lung | 10 (13%) |
| Number of metastatic sites | 1 | 29 (36%) |
|  | ≥2 | 51 (64%) |
| Measurable lesion | Yes | 73 (91%) |

EBV, Epstein-Barr virus; MMR, mismatch repair; PS, Eastern Cooperative Oncology Group performance status.
