# Supplementary material for: Clinicopathological and molecular features of responders to nivolumab for patients with advanced gastric cancer
Source: J Immunother Cancer. 2019 Jan 31;7:24. doi: 10.1186/s40425-019-0514-3 (PMC6357506; doi:10.1186/s40425-019-0514-3)
Supplement: Supplementary file 3 — Figure S1. Response to nivolumab by tumor mutation burden. (DOCX 22 kb) [file 40425_2019_514_MOESM3_ESM.docx]

Figure S1. Response to nivolumab by tumor mutation burden

Mb, megabase.
