# Supplementary material for: Clinicopathological and molecular features of responders to nivolumab for patients with advanced gastric cancer
Source: J Immunother Cancer. 2019 Jan 31;7:24. doi: 10.1186/s40425-019-0514-3 (PMC6357506; doi:10.1186/s40425-019-0514-3)
Supplement: Supplementary file 4 — Table S3. Clinicopathological features of responders to nivolumab in patients with MMR-P. (DOCX 16 kb) [file 40425_2019_514_MOESM4_ESM.docx]

Table S3. Clinicopathological features of responders to nivolumab in patients with MMR-P

| N = 60 |  | All | Responder | Non-responder | ORR | *P*-value |
| --- | --- | --- | --- | --- | --- | --- |
| Age | <65 | 20 (33%) | 4 | 16 | 20% | 0.28 |
|  | $\geq$65 | 40 (67%) | 4 | 36 | 10% |  |
| Gender | Male | 47 (78%) | 6 | 41 | 13% | 0.81 |
|  | Female | 13 (22%) | 2 | 11 | 15% |  |
| ECOG PS | 0 | 36 (60%) | 8 | 28 | 22% | 0.01 |
|  | $\geq$1 | 24 (40%) | 0 | 24 | 0% |  |
| Histology | Intestinal | 26 (43%) | 2 | 24 | 8% | 0.26 |
|  | Diffuse | 34 (57%) | 6 | 28 | 18% |  |
| Borrmann classification | Type4 | 5 (8%) | 0 | 5 | 0% | 0.36 |
|  | Others | 55 (92%) | 8 | 47 | 15% |  |
| Number of previous chemotherapy | 2 | 24 (40%) | 5 | 19 | 21% | 0.16 |
|  | $\geq$3 | 36 (60%) | 3 | 33 | 8% |  |
| Site of metastasis | Lymph node | 48 (80%) | 8 | 40 | 17% | 0.13 |
|  | Peritoneum | 30 (50%) | 3 | 27 | 10% | 0.45 |
|  | Liver | 29 (48%) | 4 | 25 | 14% | 0.92 |
|  | Lung | 10 (17%) | 1 | 9 | 10% | 0.73 |
| Number of metastatic sites | 1 | 16 (27%) | 1 | 15 | 6% | 0.33 |
|  | $\geq$2 | 44 (73%) | 7 | 37 | 16% |  |

PS, Eastern Cooperative Oncology Group performance status; MMR-P, mismatch repair proficient; ORR, objective response rate.
