# Supplementary material for: Clinicopathological and molecular features of responders to nivolumab for patients with advanced gastric cancer
Source: J Immunother Cancer. 2019 Jan 31;7:24. doi: 10.1186/s40425-019-0514-3 (PMC6357506; doi:10.1186/s40425-019-0514-3)
Supplement: Supplementary file 5 — Table S4. Molecular features of responders to nivolumab in patients with MMR-P. (DOCX 16 kb) [file 40425_2019_514_MOESM5_ESM.docx]

Table S4. Molecular features of responders to nivolumab in patients with MMR-P

|  | Assessed | Detected | Responder | Non-responder | ORR | *P*-value |
| --- | --- | --- | --- | --- | --- | --- |
| HER2+ | 60 | 16 (27%) | 1 | 15 | 6% | 0.33 |
| PD-L1+ in tumor cell (TC) | 53 | 9 (17%) | 4 | 5 | 44% | <0.01 |
| CPS$\geq$10 | 53 | 13 (25%) | 3 | 10 | 23% | 0.35 |
| CPS$\geq$1 | 53 | 47 (89%) | 8 | 39 | 17% | 0.27 |
| CPS<1 (negative) | 53 | 6 (11%) | 0 | 6 | 0% | 0.27 |
| EBV+ | 60 | 4 (7%) | 1 | 3 | 25% | 0.48 |
| TMB$\geq$10 | 47 | 26 (59%) | 4 | 22 | 15% | 0.55 |
| *ARID1A* mutation | 45 | 5 (11%) | 1 | 4 | 20% | 0.64 |
| *ERBB2* mutation | 45 | 2 (4%) | 0 | 2 | 0% | 0.57 |
| *KRAS* mutation | 45 | 2 (4%) | 0 | 2 | 0% | 0.57 |
| *MET* mutation | 45 | 1 (2%) | 0 | 1 | 0% | 0.69 |
| *PIK3CA* mutation | 45 | 4 (9%) | 1 | 3 | 25% | 0.47 |
| *TP53* mutation | 45 | 26 (58%) | 4 | 22 | 15% | 0.64 |
| *CCNE1* amplification | 45 | 7 (16%) | 2 | 5 | 29% | 0.20 |
| *ERBB2* amplification | 45 | 9 (20%) | 0 | 9 | 0% | 0.19 |
| *FGFR* amplification | 45 | 3 (7%) | 0 | 3 | 0% | 0.48 |
| *MDM2* amplification | 45 | 2 (4%) | 0 | 2 | 0% | 0.57 |
| *MYC* amplification | 45 | 3 (7%) | 0 | 3 | 0% | 0.48 |

CPS, combined positive score; EBV, Epstein-Barr virus; MMR-P, mismatch repair proficient; ORR, objective response rate, PD-L1, programmed cell death-1 ligand-1; TMB, tumor mutation burden.
