# Supplementary material for: Clinicopathological and molecular features of responders to nivolumab for patients with advanced gastric cancer
Source: J Immunother Cancer. 2019 Jan 31;7:24. doi: 10.1186/s40425-019-0514-3 (PMC6357506; doi:10.1186/s40425-019-0514-3)
Supplement: Supplementary file 6 — Table S5. Characteristics of patients with MMR-D and EBV positive gastric cancer. (DOCX 18 kb) [file 40425_2019_514_MOESM6_ESM.docx]

Table S5. Characteristics of patients with MMR-D and EBV positive gastric cancer

| MMR | EBV | Response | PS | Genomic alteration | | | PD-L1 in TC | CPS$\geq$10 | CPS$\geq$1 | Site of metastases |
| --- | --- | --- | --- | --- | --- | --- | --- | --- | --- | --- |
|  |  |  |  | mutation | amplification | TMB/Mb |  |  |  |  |
| MMR-D | - | + | 0 | NE | NE | - | - | + | + | Lymph node |
| MMR-D | - | + | 0 | NE | NE | - | + | + | + | Lymph node |
| MMR-D | - | + | 0 | *PIK3CA, TP53* | None | 38.3 | + | - | + | Lymph node, Subcutaneous |
| MMR-D | - | + | 0 | *PIK3CA* | None | 11.5 | - | - | + | Lymph node, Peritoneum |
| MMR-D | - | + | 1 | None | None | 7.7 | + | + | + | Lymph node, Peritoneum |
| MMR-D | - | + | 0 | *PIK3CA, TP53, MET* | None | 58.0 | + | - | + | Lymph node |
| MMR-D | - | - | 1 | *TSC1, PIK3CA, KRAS* | None | 19.1 | + | + | + | Lymph node |
| MMR-D | - | - | 2 | *PTEN, PIK3CA, KRAS, FBXW7* | None | 46.0 | NE | NE | NE | Liver, Subcutaneous |
| MMR-P | ＋ | + | 0 | *TP53* | None | 7.7 | - | - | + | Liver, Brain |
| MMR-P | + | - | 0 | None | *CSNK2A1, FLT3, ZNF217* | 0 | - | - | + | Liver, Peritoneum |
| MMR-P | ＋ | - | 0 | *TP53, PIK3CA, FBXW7* | *MYC* | 26.8 | - | - | + | Liver |
| MMR-P | ＋ | - | 0 | None | None | - | - | - | + | Lymph node |

CPS, combined positive score; EBV, Epstein-Barr virus; MMR, mismatch repair, MMR-D, mismatch repair deficient; MMR-P, mismatch repair proficient; NE, not examined; ORR, objective response rate, PD-L1, programmed cell death-1 ligand-1; TMB, tumor mutation burden.
