# Supplementary material for: Clinicopathological and molecular features of responders to nivolumab for patients with advanced gastric cancer
Source: J Immunother Cancer. 2019 Jan 31;7:24. doi: 10.1186/s40425-019-0514-3 (PMC6357506; doi:10.1186/s40425-019-0514-3)
Supplement: Supplementary file 7 — Table S6. Subgroup analysis of progression-free survival. (DOCX 16 kb) [file 40425_2019_514_MOESM7_ESM.docx]

Table S6. Subgroup analysis of progression-free survival

|  | | n | mPFS | Univariate analysis | | HR adjusted by PS | |
| --- | --- | --- | --- | --- | --- | --- | --- |
|  |  |  |  | HR | P-value | HR | P-Value |
| ECOG PS | 0 | 47 | 3.0 | 0.3 (0.2-0.5) | <0.01 |  |  |
|  | ≥1 | 33 | 1.1 | Reference |  |  |  |
| CPS>10 | + | 17 | 3.9 | 0.6 (0.3-1.2) | 0.14 | 0.5 (0.2-1.0) | 0.07 |
|  | - | 48 | 2.0 | Reference |  | Reference |  |
| PD-L1+ in TC | + | 15 | N.R. | 0.4 (0.2-1.0) | 0.03 | 0.4 (0.2-0.9) | 0.03 |
|  | - | 50 | 1.9 | Reference |  | Reference |  |
| MMR | MMR-D | 8 | N.R. | 0.2 (0.06-0.7) | <0.01 | 0.2 (0.0.5-0.6) | <0.01 |
|  | MMR-P | 66 | 1.8 | Reference |  | Reference |  |
| TMB | ≥10 | 34 | 1.4 | 1.4 (0.8-2.5) | 0.3 | 1.4 (0.8-2.6) | 0.2 |
|  | <10 | 24 | 2.3 | Reference |  | Reference |  |
| *PIK3CA* mutation | + | 9 | 3.9 | 0.5 (0.2-1.3) | 0.2 | 0.6 (0.2-1.3) | 0.2 |
|  | - | 47 | 2.0 | Reference |  | Reference |  |

CPS, combined positive score; HR, hazard ratio; MMR, mismatch repair; MMR-D, mismatch repair deficient; MMR-P, mismatch repair proficient; mPFS, median progression free survival; N.R., not reached; ORR, objective response rate, PD-L1+ in TC, programmed cell death-1 ligand-1 expression positive in tumor cell; TMB, tumor mutation burden.
